# Comments on “Detection and identification of enteroviruses circulating in children with acute gastroenteritis in Pará State, Northern Brazil (2010–2011)”

**DOI:** 10.1186/s12985-021-01602-3

**Published:** 2021-06-30

**Authors:** Adriana Luchs

**Affiliations:** Enteric Disease Laboratory, Adolfo Lutz Institute, Virology CenterAv. Dr Arnaldo, nº 355, São Paulo, SP 01246-902 Brazil

**Keywords:** Enterovirus, Acute gastroenteritis, Brazil

## Abstract

Investigation of human enterovirus (EV) in diarrheic fecal specimens is valuable to address EV diversity circulating worldwide. However, the detection of EV strains exclusively in fecal specimens must be interpreted cautiously. EV are well known causative agents associated with a spectrum of human diseases, but not acute gastroenteritis. EV isolation in stool samples could not necessarily be associated with diarrheic symptoms, as most EV infections appear to be asymptomatic, and healthy children could excrete EV in their stool. The diagnostic of EV is only confirmed when the neutralization test presents a significant increase in antibody titers (three times or more) in the paired serum samples (acute-phase and convalescent-phase) against the same EV serotype isolated in feces. In addition, patients suffering from acute gastroenteritis, even during an EV investigation, must be screened in parallel for gastroenteric viruses (i.e. norovirus and rotavirus) in order to clarify if the symptoms could be linked to other viral agent detected in their fecal samples. Surveillance of EV diversity among distinct patient groups, including diarrheic individuals, must be taken into consideration and can considerably increase the power of non-polio EV surveillance system in Brazil. More well-designed studies are necessary to further elucidate the role of EV in acute gastroenteritis.

## Background

Dr. Adriana Luchs is a researcher at the Enteric Virus Laboratory, Adolfo Lutz Institute, São Paulo, Brazil. Her research interests are in enteric viral pathogens.

I read with interest Machado et al. [1] investigation of human enterovirus (EV) associated to acute gastroenteritis in children from the state of Pará, Northern Brazil. This study would be valuable to formulate future plans to screen diarrheic fecal specimens in order to address EV diversity circulating in the country as well as worldwide. Nevertheless, I would like to share particular apprehensions and clarify some topics aiming to contribute to the knowledge of EV epidemiology.

The family *Picornaviridae* has undergone a significant expansion in recent years, due principally to the identification of previously unknown picornaviruses by next-generation sequencing of clinical and environmental samples [2]. In fact, these new members of *Picornaviridae* (i.e. Cosavirus and Parechovirus) have been detected in human fecal specimens, and studies have suggested their association with gastroenteritis infection [3, 4]. Nevertheless, the pathogenicity of new *Picornaviridae* members in humans remains unknown. On the other hand, EVs (*Enterovirus* genus, *Picornaviridae* family) are well known causative agents associated with a spectrum of human diseases, including acute febrile illness, hand-foot-and-mouth disease, acute flaccid paralysis, aseptic meningitis, encephalitis, myocarditis, acute respiratory syndrome and neonatal sepsis, but not acute gastroenteritis [5, 6]. Indeed, none of the studies cited [7–9] by Machado et al. [1] stated the association of EV with acute diarrhea, including those one from Brazil [10, 11].

Employing diarrheal stools to describe and investigate EV strain diversity is definitely valid, however the authors cannot declare that EV are the causing pathogen, especially without clinical/epidemiological evaluation of the patients. Attempts to associate EV as causative agent of acute gastroenteritis are not rare [12], however the detection of EV in stool could not necessarily be associated with diarrheic symptoms, as most EV infections appear to be asymptomatic [13], and over 10% of healthy children excrete EV in their stool [14]. In addition, EVs colonize the throat and gut for weeks to months and can exhibit a lingering shedding in feces after previous infections [15]. Therefore, detection of EV exclusively in these sites must be interpreted cautiously [16]. In case of suspected EV infection, multiple samples should be collected from different sites, and the samples collection are performed following the clinical symptoms [16]. Fecal specimens alone are not the suitable for EV diagnosis. Stool samples must be accompanied by paired blood samples (acute-phase and convalescent-phase). The diagnostic of EV is only confirmed when serological tests (neutralization test) present a significant increase in antibody titers (three times or more) in the paired serum samples against the same EV serotype isolated in feces [16, 17].

At least, it is important to mention that the EV strains detected in Machado et al. [1] study were identified in fecal specimens from children suffering from acute gastroenteritis. The 175 stool samples very likely were screened for gastroenteric viruses (i.e. norovirus and rotavirus) following the algorithm of clinical symptoms at the Pediatric Clinic of Pará. The authors failed to present these data in the manuscript. As EV are classically not known to be associated with acute diarrhea [5, 6], the gastroenteritis symptoms observed in the patients could be linked to one (or more) of the gastroenteric viruses eventually detected in their fecal samples.

## Conclusion

Identifying the circulating EV strains in Brazilian inhabitants is vital to elucidate the enteroviral biodiversity in the country and improve our understanding of their potential health burden, especially considering the capacity of EV to remain in silent circulation in populations [11]. Surveillance of EV diversity among distinct patient groups, including diarrheic individuals, must be taken into consideration and can considerably increase the power of non-polio EV surveillance system in Brazil. More well-designed studies are necessary to further elucidate the role of EV in acute gastroenteritis.

## Data Availability

Not applicable.

## Abbreviation

EV: Enterovirus
